# Habituation to Pain in Patients with Chronic Pain: Clinical Implications and Future Directions

**DOI:** 10.3390/jcm12134305

**Published:** 2023-06-27

**Authors:** Maite M. van der Miesen, Catherine J. Vossen, Elbert A. Joosten

**Affiliations:** 1 Department of Anesthesiology and Pain Management, School for Mental Health and Neuroscience (MHeNS), Faculty of Health, Medicine and Life Sciences (FHML), Maastricht University, 6229 ER Maastricht, The Netherlands; c.vossen@mumc.nl (C.J.V.); bert.joosten@mumc.nl (E.A.J.); 2 Department of Anesthesiology and Pain Medicine, Maastricht University Medical Centre, 6229 HX Maastricht, The Netherlands

**Keywords:** chronic pain, habituation, sensitization, migraine, fibromyalgia, chronic low back pain

## Abstract

In this review, the latest insights into habituation to pain in chronic pain are summarized. Using a systematic search, results of studies on the evidence of habituation to (experimental) pain in migraine, chronic low back pain, fibromyalgia, and a variety of chronic pain indications are presented. In migraine, reduced habituation based on self-report and the EEG-based N1 and N2–P2 amplitude is reported, but the presence of contradictory results demands further replication in larger, well-designed studies. Habituation to pain in chronic low back pain seems not to differ from controls, with the exception of EEG measures. In fibromyalgia patients, there is some evidence for reduced habituation of the N2–P2 amplitude. Our analysis shows that the variability between outcomes of studies on habituation to pain is high. As the mechanisms underlying habituation to pain are still not fully understood and likely involve several pathways, it is now too early to conclude that habituation to pain is related to clinical outcomes and can be used as a diagnostic marker. The review ends with a discussion on future directions for research including the use of standard outcome measures to improve comparisons of habituation to pain in patients and controls, as well as a focus on individual differences.

## 1. Introduction

Habituation is a simple non-associative form of learning that is defined as a response decrement resulting from repeated stimulation, which does not involve sensory adaptation or motor fatigue [1]. Habituation has been reported for numerous stimuli such as auditory, visual, and sensory and has been measured in humans using reflexes, ratings, and physiological measures such as skin conductance, electroencephalography (EEG), and functional magnetic resonance imaging (fMRI) [2]. In addition to habituation, sensitization to repetitive stimulation might occur, which is defined as an increase in response [1,3]. In the field of pain, habituation is usually studied using external stimuli such as heat or electrical current [4]. 

Reduced habituation has been suggested to occur in a number of neuropsychiatric disorders such as autism [2]. For chronic pain, however, this is not yet well established. Although deficits in habituation may occur in relation to chronic pain, most research in the field has focused on sensitization processes, especially central sensitization. Central sensitization is defined by the International Association for the Study of Pain (IASP) as an increased responsiveness of the central nervous system to normal or subthreshold input [5]. Central sensitization is thought to be implicated in several chronic pain disorders such as fibromyalgia (see, e.g., [6]) and is characterized by hyperalgesia (increase in sensitivity) and allodynia (pain due to a stimulus that would normally not cause pain). Interestingly, central sensitization is often studied based on the cellular level. Recently, researchers argued that the behavioral approach, such as measuring pain ratings as an outcome, should be more emphasized in research on sensitization to pain [7]. Notably, in this review, when discussing sensitization, we refer to an increase in pain on the behavioral level. 

For both healthy individuals and chronic pain patients, the mechanism of habituation and sensitization to pain is not fully understood. Several theories have been proposed such as the dual-process theory [8]. This theory states that habituation and sensitization processes may interact to produce the behavioral outcome [8]. One recent proposed mechanism is stimulus-dependent feedback inhibition or inhibitory potentiation, which decreases incoming stimuli [9]. Prior experience, thus, affects the firing of neurons. This mechanism can be seen as a form of predictive coding [9].

Reduced habituation to pain in chronic pain patients has been mainly reported in the indications migraine, chronic low back pain (CLBP), and fibromyalgia [4]. Although numerous studies have been published, no review of the literature is, to our knowledge, available. The main aim of this review is, therefore, to summarize the literature on habituation to pain based on effects of repeated painful stimulation in chronic pain patients (with a focus on migraine, CLBP, and fibromyalgia) as well as its potential treatment targets and clinical implications. We hope that this review may serve as a knowledge basis to design new innovative studies on habituation to pain in chronic pain.

In this review, results of patients versus controls are discussed. When comparing two groups with respect to habituation, several terms are used such as altered, decreased, or impaired habituation. McDiarmid and colleagues (2017) formulated recommendations to interpret the responses of repeated stimulation, i.e., habituation curves or trajectories [2]. In the literature included in the review, however, these recommendations have not been fully implemented, and a quantifiable measure of habituation is not available. For the current review, therefore, we used the term *reduced* habituation to pain if patients showed less habituation following repeated painful stimulation as compared to controls (i.e., the decrease in VAS was less than in controls). Furthermore, reduced habituation in patients may also include and be indicative for sensitization to pain. If available, the direction of the effect, i.e., whether patients and/or controls showed habituation, no change, or sensitization to pain, is discussed and presented in tables.

## 2. Materials and Methods

The search was preregistered at the Open Science Framework (osf.io/nypbw). Articles were selected using a systematic search of PubMed, PsycINFO, and Web of Science databases (up until January 2023). For extensive search criteria and the selection procedure of included articles, see the Supplementary Materials (Figure S1). The articles included from our systematic search were split into those investigating healthy individuals (revised manuscript submitted) and chronic pain patients, which are the focus of this review. 

## 3. Results

**Figure 1:**
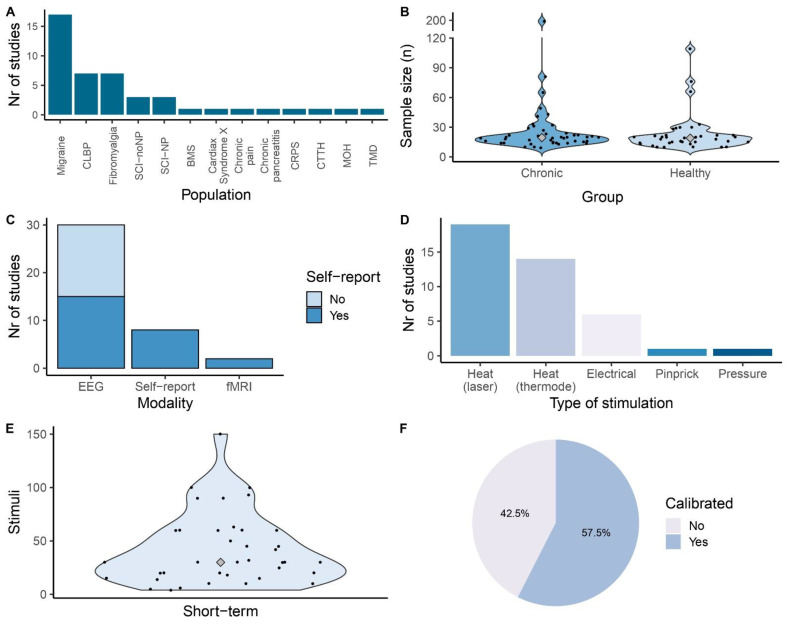
Overview of included studies. Gray diamonds indicate the median. (**A**) Number of studies per chronic pain indication. (**B**) Sample sizes for chronic and healthy populations. (**C**) Number of studies per modality and whether they included self-report. (**D**) Type of stimulation used. (**E**) Number of stimuli to measure habituation. (**F**) Use of individual calibration in studies. BMS, burning mouth syndrome; CLBP, chronic low back pain; CRPS, complex regional pain syndrome; CTTH, chronic tension-type headache; EEG, electroencephalography; fMRI, functional magnetic resonance imaging; MOH, medication-overuse headache; SCI-NP, spinal cord injury with neuropathic pain; SCI-noNP, spinal cord injury without neuropathic pain; TMD, temporomandibular disorder.

The systematic database search of PubMed, PsycINFO, and Web of Science resulted in the inclusion of *n* = 40 articles. The results of this search showed that most studies were performed in patients with headache disorders (mostly migraine, *n* = 17), CLBP (*n* = 7), and fibromyalgia (*n* = 7) (see Figure 1A). Therefore, we subdivided these sections accordingly. Sample sizes varied greatly between nine and 199 included participants (Figure 1B). Most research included self-report ratings of pain (*n* = 25), whereas EEG was the most used method (*n* = 30) followed by solely self-report ratings (*n* = 8) (Figure 1C). Heat stimuli using a thermode and heat stimuli using a laser were most frequently used for repeated painful stimulation (Figure 1D). Only two studies investigated long-term habituation to pain [10,11], whereas all other studies investigated short-term habituation to pain. For this, a wide range of stimulus repetitions were used, with a median of 30 (Figure 1E). The majority of these studies performed individual calibration to decide on the stimulus intensity, whereas 42.5% of the studies used a fixed intensity level (Figure 1F). 

### 3.1. Headache Disorders

**Figure 2:**
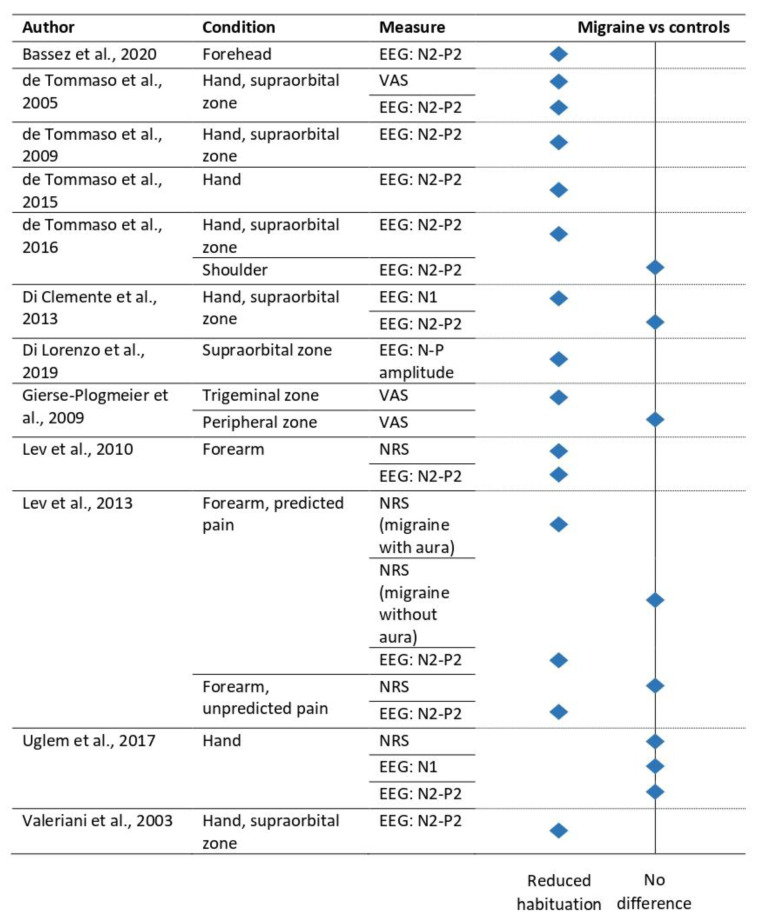
Effect of habituation to pain in migraine patients versus controls. Note: Only those studies using direct group comparisons are included. EEG, electroencephalography; NRS, numeric rating scale; VAS, visual analogue scale [14,15,17,18,19,20,22,23,24,25,29].

Headache disorders are among the most common chronic central nervous system disorders, with migraine being the indication most studied [12]. In migraine research, several research lines have focused on habituation to sensory stimuli (e.g., visual, auditory, and painful responses) [13]. It has been hypothesized that reduced habituation to pain in migraine patients may be caused by increased cortical excitability, decreased inhibition, or decreased pre-activation levels [14]. Data on habituation to pain using self-report and EEG, with a focus on migraine patients are presented in Figure 2 and Table 1.

For self-report using electrical stimuli, reduced habituation was reported at the trigeminal area but not at the tibial region [15]. Other studies that included different stimulation sites showed similar habituation for self-report at different stimulation sites [16,17]. Studies using heat stimuli showed mixed effects: habituation in both patients and in controls [16], reduced habituation in patients with migraine with aura for predicted pain [18], or reduced habituation in patients without aura [19]. Interestingly, a large study using laser stimuli showed no self-reported habituation differences between migraine patients and controls [20], which contrasts with earlier findings [17,21]. 

Results from evoked potentials with heat stimuli showed similar habituation [16] or reduced habituation in patients versus controls [18,19]. Several studies focused on laser-evoked potentials (LEPs) and reported a reduced N2–P2 amplitude habituation in migraine patients [17,21,22,23,24]. Another study reporting evidence for reduced habituation of the N2–P2 amplitude showed that this effect over time was accompanied by increased connectivity between the thalamus and somatosensory areas in migraine patients, but not in controls [14]. Contradictorily, an observer-blinded longitudinal study with large sample sizes (*n* = 30–49) using advanced statistical models did not report any group differences and noted similar habituation of the N2–P2 amplitude for migraine patients and controls [20]. The authors of the latter study provided a detailed comparison of studies using LEP’s in migraine patients and argued that the evidence for reduced habituation in migraine patients is low [20]. Results for N1 amplitude habituation are less frequently reported and also mixed, with two studies reporting differences [23,25], although the latter did not compare groups directly, and one study reported no group differences [20]. 

Furthermore, it was shown that LEP amplitudes did not differ between chronic tension-type headache (CTTH) patients and healthy controls, although migraine patients showed reduced habituation compared to CTTH patients [23]. Additionally, the LEP amplitudes were not influenced by migraine phase [17,20], visually induced analgesia [26], or the presence or absence of aura [20]. Menstrual phase affected the amount of habituation in both migraine patients and healthy controls [24]. 

In summary, taking into account the large variability in study design, parameters, and outcome measures, it is tentatively concluded that there is only limited evidence that migraine patients show reduced habituation for both self-report and the N1 and N2–P2 amplitude. This conclusion takes into consideration that several studies reported contradictory effects (see Figure 2), and one blinded study with a large sample size did not report differences [20]. More conclusive evidence is needed, and this should be based on large-scale randomized study designs.

**Table 1:** Overview of habituation to pain in chronic headache disorders. ↘: decrease over time (habituation). →: no significant change. ↗: increase over time (sensitization). <: reduced habituation in the chronic pain patient compared to the control group. =: no significant difference in habituation between the groups. C, control; CHEP, contact heat-evoked potential; CTTH, chronic tension-type headache; EEG, electroencephalography; ISI, inter-stimulus interval; M, migraine; MOA, migraine without aura; MOH, medication-overuse headache; MWA, migraine with aura; NRS, numeric rating scale; PREP, pain-related evoked potential; sLORETA, standardized low-resolution brain electromagnetic tomography; VAS, visual analogue scale.

	Sample Size	Timescale	Type of Stimuli	Site	Nr of Stimuli for Habituation Analysis	Duration	ISI	Habituation Measurement	HabituationAnalysis	Main Habituation Result
**Bassez et al., 2020** [14]	M = 23 C = 20	Short-term	Heat (CO_2_ laser)	Right forehead	15	Intensity (and, thus, duration) varied per participant, 15–45 ms	Self-paced, ± 10 s	EEG: N2–P2 amplitude Dynamic causal modelling	% change between first and third block Connectivity changes over blocks	M < C Increased thalamo-somatosensory connectivity in migraine patients
**Beese et al., 2015** [16]	M = 22 (12 with aura) C = 22	Short-term	Heat (thermode)	Volar forearm, left and right cheek	20	-	15–18 s	NRS: single trial CHEP: N2–P2 amplitude	Average of first 5 trials vs. last 5 trials Average of first 5 trials vs. last 5 trials	NRS for each site and block: M ↘ C ↘ N2–P2 for each site and block: M ↘ C ↘
**de Tommaso et al., 2005** [17]	M = 14 (without aura) C = 10	Short-term	Heat (CO_2_ laser)	Right and left hand and supraorbital zone	60	20 ms	10 s	NRS: single trial EEG: N2–P2 amplitude	Trend over block Trend over block	NRS both hand and face: M ↗ < C ↘ N2-P2 both hand and face: M → < C ↘ No differences between migraine phase
**de Tommaso et al., 2005** [21]	M = 14 (without aura) C = 10	Short-term	Heat (CO_2_ laser)	Right supraorbital zone	63	20 ms	10 s	VAS: single trial EEG: N2–P2 amplitude	Trend over block Trend over block	VAS: M → C ↘ N2-P2: M → C ↘
**de Tommaso et al., 2009** [24]	M = 9 (without aura) C = 10	Short-term	Heat (CO_2_ laser)	Right dorsum of the hand and supraorbital zone	60	25 ms	10–15 s	EEG: N2–P2 amplitude	Ratio of amplitude between block 1 and 3	Both hand and face: M < C Menstrual cycle affects habituation of N2–P2
**de Tommaso et al., 2015** [22]	M = 31 (without aura) C = 19	Short-term	Heat (CO_2_ laser)	Dorsum of the right hand	30	30 ms	10 s	EEG: N2–P2 amplitude	Stimuli were divided in three blocks, % change relative to first block	M < C
**De Tommaso et al., 2016** [27]	M = 20 C = 20	Short-term	Heat (CO_2_ laser)	Dorsum of the right hand, right supraorbital zone and the skin over the right trapezius	30	30 ms	7 s	EEG: N2–P2 amplitude	Stimuli were divided in three blocks, % change relative to first block	Baseline N2–P2 hand and face: M < C Shoulder: M = C After onabotulintoxin A treatment, N2–P2 habituation at face increased, no effect on hand and shoulder habituation
**De Tommaso et al., 2021** [28]	M = 17	Short-term	Heat (CO_2_ laser)	Dorsum of the right hand and left and right supraorbital zone	30	30 ms	10 s	EEG: N1, N2, P2 amplitude	Ratio between average of last and first 10 stimuli	No effect of erenumab on N1 and P2 at both sides, increased habituation after erenumab at N2 of left forehead
**Di Clemente et al., 2013** [25]	M = 13 (without aura) C = 15	Short-term	Heat (YAP laser)	Dorsum of the right hand and right supraorbital zone	45	-	10 s	EEG: N1 and N2–P2 amplitude	% change between first and third block	N1 hand and face: M < C N2–P2 hand and face: M = C Topiramate reduces N1 habituation deficit/affects habituation
**Di Lorenzo et al., 2019** [29]	M = 18 (without aura) C = 18	Short-term	Electrical	Right supraorbital notch	10	Train of 3 0.1 ms pulses with 5 ms interal (total 10.3 ms)	30–35 s	PREP: N–P amplitude	Slope between first and second block	Before treatment: M < C After treatment: no group comparison Ketogenic diet increased habituation in migraine
**Ferraro et al., 2012** [30]	MOH = 14 (group I treatment effective = 8; group II treatment not effective = 6) C = 14	Short-term	Heat (CO_2_ laser)	Dorsum of the right hand and perioral region	90	10 ms	8–12 s	VAS: after each block EEG: N1 and N2–P2 amplitude	Percentage of the first block Percentage of the first block	VAS: Before treatment for hand and face: MOH group I and II < C After treatment hand: MOH group II < C and MOH group I After treatment face: MOH group I and II = C EEG: N1 hand before treatment: MOH group I and II < C N1 hand after treatment: MOH group I and II = C N1 face before treatment: MOH group II < C N1 face after treatment: MOH group I and II < C N2–P2 hand and face before treatment: MOH group I and II < C N2–P2 hand and face after treatment: MOH group II < C and MOH group I Treatment affected habituation in clinically improved patients
**Gierse-Plogmeier et al., 2009** [15]	M = 20 (10 with aura) C = 20	Short-term	Electrical	Trigeminal (masseter region), peripheral (tibial region)	20	1 ms	2 s	VAS: last stimulus of train	Group comparison of difference score between trains	Trigeminal: M < C Peripheral: M = C
**Lev et al., 2010** [19]	M = 21 (with aura) C = 22	Short-term	Heat (thermode)	Left volar forearm	60	-	–	NRS: single trial CHEP: N2–P2 amplitude and sLORETA	Group comparison of inter-train changeGroup comparison of inter-train change	NRS: M ↗ < C → N2-P2: M ↗ < C ↘ > M < C activity in contralateral orbitofrontal cortex M > C in contralateral primary somatosensory cortex, insula, parahippocampal cortex, and bilateral posterior cingulate cortex
**Lev et al., 2013** [18]	MWA = 20MOA = 19 C = 22	Short-term	Heat (thermode)	Left volar forearm	50	-	10 s	NRS: single trial CHEP: N2–P2 amplitude and sLORETA	Group comparison of inter-train change Group comparison of inter-train change	NRS: Predicted pain: MWA ↗ < C → and MOA → Unpredicted pain: MOA ↗ = MWA ↗ = C → N2-P2: Predicted pain: MOA → and MWA ↗ < C ↘ Unpredicted pain: MOA ↗ and MWA ↗ < C → Predicted pain: MWA < C activity in right inferior frontal gyrus and supplementary motor area MWA > C activity in primary and secondary somatosensory cortex, motor cortex, and bilateral posterior cingulate cortex MOA > C activity in right insula Unpredicted pain: MWA > C activity in bilateral medial frontal cortex, right anterior cingulate cortexMOA > C activity in right motor cortex, primary and secondary somatosensory cortex, left orbitofrontal cortex, parahippocampal cortex, and insula
**Sava et al., 2018** [26]	M = 14 (without aura) C = 11	Short-term	Heat (thermode)	Right volar wrist or forehead	20	707 ms	10–22 s	CHEP: P1–P2 slope	Slope over average of 5 blocks	Mirror did not influence habituation in controls or migraine patients
**Sebastianelli et al., 2023** [31]	M = 15	Short-term	Electrical	Supraorbital nerve at the forehead	18	Three 0.1 ms pulses with 5 ms interval	40 s	EEG: N–P amplitude	Slope of the amplitude between the first and third block	No effect of onabotulintoxin A on habituation
**Uglem et al., 2017** [20]	M = 49 (27 without aura, 4 with, 18 both) C = 30	Short-term	Heat (YAP laser)	Dorsum of the right hand	42	6 ms	6–10 s	NRS: single trial EEG: N1 and N2–P2 amplitude	Multilevel models	NRS: M → = C → N1: M → = C → N2-P2: M ↘ = C ↘ Habituation was mainly similar between migraine phases
**Valeriani et al., 2003** [23]	M = 24 (without aura) CTTH = 19 C = 28	Short-term	Heat (CO_2_ laser)	Left and right dorsum of the hand and face	45 for face, 90 for hand	10 ms	8–12 s	EEG: N1–P1 and N2–P2 amplitude	% decrease over blocks	N1–P1 hand: M → C ↘ N1–P1 face: M → C → N2–P2 hand: M ↘ < C ↘, CTTH ↘ = C ↘, M ↘ < CTTH ↘ N2–P2 face: M → < C ↘, CTTH ↘ = C ↘

### 3.2. Chronic Low Back Pain

Chronic low back pain (CLBP) is known for its high prevalence and large global impact on health and society [32]. The majority of CLBP patients have pain without a specific patho-anatomical cause, and this pain is, therefore, described as “nonspecific” [33]. 

**Figure 3:**
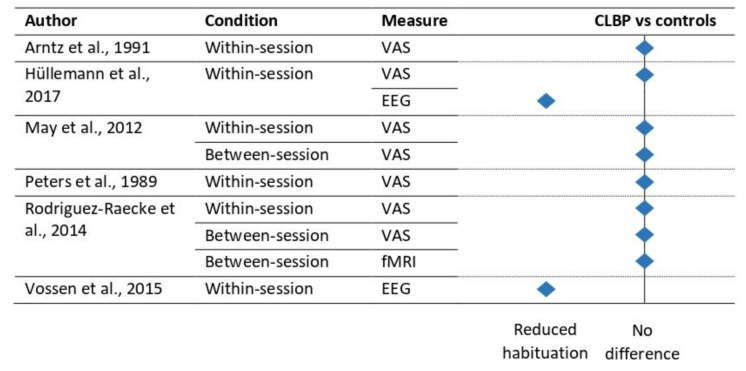
Effect of habituation to pain in CLBP patients versus controls. Note: Only those studies using direct group comparisons are included. EEG, electroencephalography; fMRI, functional magnetic resonance imaging; VAS, visual analogue scale [10,11,34,35,36,37].

The available literature for habituation to pain in CLBP patients is relatively limited, yet still diverse (see Figure 3 and Table 2). Early short-term studies using pressure or electrical stimuli did not demonstrate any self-report differences between CLBP patients and controls, independent of the presence [34] or absence [35] of habituation to pain. CLBP patients (diverse pain population and short disease duration) and controls reported similar (long-term) habituation to pain over time, as well as within-session sensitization to pain with use of an 8 day heat paradigm [10,11]. No changes in brain activity related to differences in habituation were shown between CLBP patients and controls, both over days or within sessions [11]. In patients with painful radiculopathy, LEP habituation was reduced, although this effect was not apparent for pain ratings [36]. Two studies adopted newer analysis methods for the study of habituation. Vossen et al. (2015) explored the EEG-amplitude signal at a very detailed scale, partitioning the post-stimulus epoch in 20 ms areas under the curve (event-related fixed-interval areas; ERFIAs) in combination with multilevel modeling [37]. Reduced habituation to pain was reported in CLBP patients at 340 to 460 ms post stimulus after painful stimuli [37]. The applicability of high temporal resolution analysis of LEP signals and habituation in radiculopathy patients was shown to be limited as a result of the data quality [38].

Both short- and long-term habituation to pain in CLBP patients did not seem to differ from that noted in controls according to self-report and long-term fMRI studies (Figure 3). Temporarily restricted effects, as measured with EEG, however, were shown to effect habituation to pain in CLBP patients. The latter needs further replication to investigate the robustness and reproducibility of this effect.

### 3.3. Fibromyalgia 

Fibromyalgia is characterized by chronic widespread pain and potential comorbidities such as disturbed sleep and psychological problems [39].

**Figure 4:**
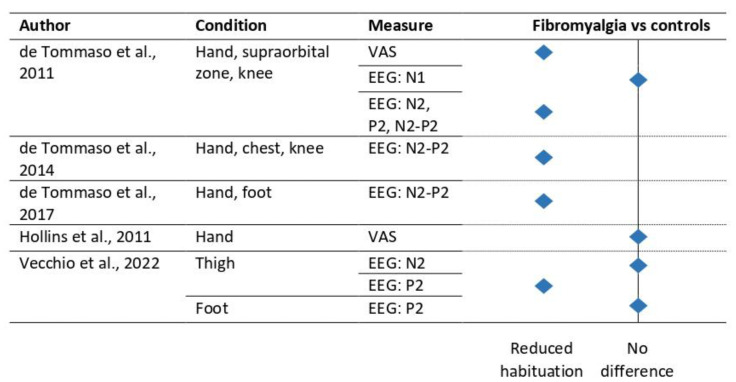
Effect of habituation to pain in fibromyalgia patients versus controls. Note: Only those studies using direct group comparisons are included. EEG, electroencephalography; VAS, visual analogue scale [40,41,42,43,44].

Hollins et al. (2011) investigated habituation to pain as a function of the time course of pain ratings in patients with fibromyalgia and controls using heat pain stimuli [40]. Both fibromyalgia patients and controls displayed first an adaptation phase followed by a sensitization phase within each run. In addition, they showed habituation to pain over the runs. The magnitude of the initial adaptation phase increased over the runs. For both the habituation (within and over runs) and the sensitization to pain, no group effects were found [40]. Conversely, de Tommaso et al. (2011) did report differences in fibromyalgia patients compared tocontrols with respect to habituation of pain ratings [41] (see Figure 4). It should be taken into account that differences in sample size, stimulus type, and the way of measuring habituation make it difficult to compare the results from de Tommaso et al. (2011) with those reported by Hollins and colleagues (2011) (see Table 3). Analyses using EEG showed reduced habituation for the N2, P2, and N2–P2 amplitude in fibromyalgia patients, but not for the N1 amplitude [41]. Follow-up EEG studies from the same laboratory reported again reduced habituation to pain of the N2–P2 amplitude in fibromyalgia patients as compared to controls [42,43]. Interestingly, in a comparative study, a more pronounced reduction in habituation to pain was shown in patients with comorbid migraine or sensory deficits as compared with fibromyalgia patients without comorbidities [42]. Habituation to pain on the thigh (for the N2 component) and foot (for the P2 component) did not result in differences between fibromyalgia patients and controls [44]. 

On the basis of the EEG studies in fibromyalgia patients, there is some evidence for reduced habituation to pain of the N2–P2 amplitude (see Figure 4). These effects demand further replication in order to infer clinical significance. Only two studies investigated self-report with contradictory findings, which needs further investigation. 

### 3.4. Other Chronic Pain Indications

In this subsection, the studies on habituation to pain related to a variety of chronic pain indications (burning mouth syndrome, temporomandibular disorder, cardia syndrome X, chronic pancreatitis, spinal cord injury-related neuropathic pain, and complex regional pain syndrome) are summarized. Temporomandibular disorder is characterized by chronic pain located in the jaw and temporomandibular joint and is a subgroup of primary orofacial pain [47,48]. The same applies to burning mouth syndrome, which may cause a chronic burning sensation in and around the mouth [49].

Using fMRI, patients with burning mouth syndrome (BMS) showed reduced brain activity in the dorsal anterior cingulate cortex (dACC), bilateral ventral midcingulate cortex (MCC), left posterior cingulate cortex (PCC), and cerebellum over the course of four thermal stimuli [50] (see Table 4). This habituation effect of brain activity was not noted in controls, who only showed increased brain activity in the PCC over time [50]. Patients with temporomandibular disorder (TMD) did not show different habituation from both controls and fibromyalgia patients [40].

In patients with cardiac syndrome X, reduced habituation was shown after laser stimulation, which was more apparent at the chest than at the hand for self-report ratings and the N2–P2 amplitude [51]. Olesen et al. (2013) investigated contact-heat evoked potentials (CHEPs) in chronic pancreatitis patients [52]. Reduced habituation for both pain ratings and the N2–P2 amplitude over time was shown and this was more pronounced for stimulation at the chest (pancreatic area) as compared to the forearm [52]. Studies based on analysis of patients with and without spinal cord injury (SCI)-related central neuropathic pain reported mixed results (see Table 4). One study demonstrated reduced habituation to pain in patients with neuropathic pain for pain ratings and CHEPs as compared to both healthy controls and to SCI patients without central neuropathic pain [53]. Conversely, absence of any difference in habituation of CHEPs or habituation of pain ratings between SCI with and without neuropathic pain were also reported [54,55]. It should be noted that SCI is characterized by its heterogeneity based on lesion size, location, and type of injury, and that this may significantly affect the development of chronic neuropathic pain in these patients. Moreover, although all three studies tested above the level of injury, Kumru et al. (2012) stimulated at the shoulder, while Albu et al. (2015) and Lütolf et al. (2022) stimulated at the hand and forearm, respectively. This variability on top of the heterogeneity of patients described above may underlie the differences in the literature on effect of habituation to pain in patients with this indication.

In patients with complex regional pain syndrome (CRPS) both heat pain ratings and pinprick ratings did not result in (reduced) habituation or differences between patients and healthy individuals [56]. A study using EEG including chronic pain patients based on a variety of indications reported no differences in pain ratings over a series of electrical stimuli [57]. Nevertheless, both the presence of chronic pain and the hypervigilance independently affected habituation of the EEG signal at several time latencies [57]. 

In summary, a trend can be noted toward reduced habituation to pain in a variety of chronic pain indications. Nonetheless, the available evidence is often based on one study for a specific pain indication and with small sample sizes. These constraints do not allow making conclusive statements about differences in habituation effects specifically related to the individual indications or to chronic pain in general.

**Table 4:** Overview of habituation to pain in other indications. ↘: decrease over time (habituation). →: no significant change. ↗: increase over time (sensitization). <: reduced habituation in the chronic pain patient compared to the control group. =: no significant difference in habituation between the groups. BOLD, blood-oxygen-level-dependent; BMS, burning mouth syndrome; C, control; CAD, coronary artery disease; CSX, cardiac syndrome X; CHEP, contact heat evoked potential; CP, chronic pancreatitis; CRPS, complex regional pain syndrome; EEG, electroencephalography; F, fibromyalgia; ISI, inter-stimulus interval; NRS, numeric rating scale; SCI-NP, spinal cord injury with neuropathic pain; SCI-noNP, spinal cord injury without neuropathic pain; TMD, temporomandibular disorder; VAS, visual analogue scale; VRS, verbal rating scale.

	Sample Size	Timescale	Type of Stimuli	Site	Nr of Stimuli for Habituation Analysis	Duration	ISI	Habituation Measurement	HabituationAnalysis	Main Habituation Result
**Albu et al., 2015** [54]	SCI-noNP = 10 SCI-NP = 10 C = 10	Short-term	Heat (thermode)	Thenar eminence of dominant hand	10	-	20 s	NRS: single trial CHEP: N2–P2 amplitude	Percentage last stimulus with respect to first stimulus	NRS and CHEP: SCI-noNP = SCI-NP = C
**Hollins et al., 2011** [40]	F = 17 TMD = 29 C = 29	Short-term	Heat (thermode)	Base of the thumb	33	3 s	12 s	VAS: single trial	Decrease over blocks	F = TMD = C
**Kumru et al., 2012** [53]	SCI-noNP = 22 SCI-NP = 32 C = 16	Short-term	Heat (thermode)	Shoulder	14	-	30 s	NRS: single trial CHEP: N2–P2 amplitude	% change of last compared to first stimulus	NRS and CHEP: SCI-NP < SCI-noNP SCI-NP < C
**Lütolf et al., 2022** [55]	SCI-noNP = 13, SCI-NP = 17, C = 14	Short-term	Heat (thermode)	Right volar forearm	10	-	15–19 s	NRS: single trial	Percentage decrease	SCI-noNP = SCI-NP = C
**Olesen et al., 2013** [52]	Chronic pancreatitis = 15 C = 15	Short-term	Heat (thermode)	Right forearm and upper abdominal area	93	-	8–12 s	VAS: first and last stimulus of block CHEP: N1 and N2–P2 amplitude	Change over blocks	Abdominal: CP → < C ↘ Forearm: CP → < C ↘ Abdominal: N1: CP = C N2–P2: CP → < C ↘ Forearm: N1: CP = C N2–P2: CP → = C ↘
**Scheuren et al., 2023** [56]	CRPS = 20, C = 16	Short-term	Heat (thermode), pinprick	Affected and control area	15	-	13–17s	NRS: single trial	% change in third compared to first block and trend over blocks	Heat: CRPS → = C → Pinprick: CRPS → = C →
**Shinozaki et al., 2016** [50]	BMS = 16 C = 15	Short-term	Heat (thermode)	Right palm and right lower lip	4	32 s	104 s	NRS: single trial fMRI: BOLD	Stimulus 1 compared to 4	NRS: Lip: BMS → C ↘ Palm: BMS → C → fMRI: Lip: Reduced activity in BMS patients over time in the right dorsal anterior cingulate cortex, bilateral ventral midcingulate cortex, left posterior cingulate cortex, right angular gyrus, and left cerebellum. Increased activity in controls over time in the left posterior cingulate cortex.
**Valeriani et al., 2005** [51]	Cardiac syndrome X = 16 Coronary artery disease = 10 C = 13	Short-term	Heat (CO_2_ laser)	Dorsum of the right hand and chest	90	10 ms	8–12 s	VAS: per block EEG: N1–P1 and N2–P2 amplitude	Trend over blocks	Chest: VAS and N2–P2: Cardiac SX → < C ↘ and CAD ↘ N1–P1: Cardiac SX = CAD = C Hand: VAS: Cardiac SX ↗ < C ↘ and CAD ↘ N1–P1: Cardiac SX = CAD = C N2–P2: Cardiac SX = CAD = C
**Vossen et al., 2018** [57]	Chronic pain (various) = 33 C = 33	Short-term	Electrical	Left middle finger	25	10 ms	9–11 s	VRS: single trial EEG: amplitude	Multilevel model with event-related fixed-interval areas	VRS: Chronic pain ↘ = C ↘ No influence of hypervigilance on pain ratings Chronic pain status and hypervigilance independently influenced the EEG-amplitude

## 4. Treatments and Clinical Implications

In this section, the clinical implications for habituation (or sensitization) to pain in chronic pain patients and potential treatments targeting (reduced) habituation are discussed. 

### 4.1. Habituation to Pain and Clinical Outcomes

Our search revealed that most studies on habituation to pain and clinical outcomes were related to fibromyalgia or migraine patients (see Table 1 and Table 3). 

In fibromyalgia, habituation was shown to be correlated with pain at tender points [42], and patients with reduced habituation showed greater widespread pain [43]. De Tommaso et al. (2011) furthermore reported a correlation between reduced habituation of the (EEG-based) N2 amplitude and self-reported depressive symptoms in fibromyalgia patients [41], although this was not replicated in a larger scale study [42]. Furthermore, an association of habituation to pain with self-reported daily activity was reported [45]. Two studies investigated the relation between EEG signal intensity and intra-epidermal nerve fiber density (IENFD) in fibromyalgia patients. Reduced habituation of the N2–P2 component [46] or P2 component [44] was reported to be related to reduced IENFD. Subgroup analysis of those fibromyalgia patients with a reduced distal IENFD revealed that the P2 component increased over time [44]. 

In migraine patients, reduced habituation at the trigeminal area was correlated with migraine attack frequency [15]. Changes in brain activity in the somatosensory cortex and parietal cortex were shown to be correlated with attacks per month, whereas orbitofrontal activity correlated with disease duration [18]. Disease duration was further correlated with reduced habituation between migraine phases based on the EEG-signal (i.e., N2–P2 amplitude) [20]. Habituation to pain did not correlate with number of days until the next attack in the migraine patients [20]. 

In conclusion, some evidence exists that cortical habituation might be linked to the severity and frequency of pain complaints in fibromyalgia or migraine patients, as well as to IENFD in fibromyalgia patients.

### 4.2. Treatments Targeting Habituation to Pain

Currently, the literature on the treatments and effects on habituation to pain is limited to headache patients only. In medication-overuse headache patients, habituation of the N2–P2 amplitude was partially restored after 6 weeks in those that had clinically improved after an acute medication withdrawal treatment [30]. These findings suggest that medication overuse aggravates symptoms by central sensitization. In another study, preventive application of topiramate, an antiepileptic drug targeting among others GABA (more inhibition) and glutamate (less excitation), normalized the habituation pattern to nociceptive stimulation in migraine patients for the N1 amplitude, but at the same time did not result in effects on habituation of the N2–P2 amplitude [25]. The authors reasoned that topiramate has an effect on the sensory-discriminative component involved in habituation to pain, i.e., the secondary somatosensory cortex. Moreover, treatment with a ketogenic diet improved habituation of electrical evoked potentials, although a comparison to controls without the diet was not available [29]. A ketogenic diet has several mechanisms of action, including enhancing GABA transmission, and increasing BDNF expression and attenuation of inflammation [58]. With respect to the N2–P2 amplitude, one study reported that onabotulintoxin A (affecting neurotransmitter release) was effective for reduced habituation to pain, but only in the trigeminal area [27,59]. Furthermore, this treatment was shown to be more effective in migraine patients with severe reduced habituation [27]. This effect was, however, not shown in a similar study using electrical stimuli [31]. Furthermore, a recent pilot study reported that Erenumab (an antibody against receptors of the nociceptive neurotransmitter calcitonin gene-related peptide (CGRP)) affected the initially reduced habituation of the N2 amplitude in migraineurs [28]. On the basis of the findings of this pilot study, further confirmation is needed based on large-scale (randomized) studies. Numerous other pharmacological options are available for chronic pain treatment, such as nonsteroidal anti-inflammatory drugs (NSAIDs), opioids, pregabalin, and selective serotonin and noradrenalin reuptake inhibitors (SNRIs) [60]. It would be interesting to investigate the effects of these treatments on habituation to pain.

### 4.3. Discussion and Future Directions

Possible treatments for habituation to pain may target different mechanisms as described above. These underlying mechanisms are complex and include various extra- and intracellular pathways. For example, topiramate has been reported to act via multiple mechanisms of action, such as the blockage of voltage-gated sodium channels, the enhancement of GABA-A receptors, the inhibition of L-type voltage-gated calcium channels, and/or the blockage of AMPA receptors [61]. These mechanisms are known to be involved in the development and maintenance of chronic pain and can be used as targets. GABA-neurotransmission is often linked to habituation of cellular processes in the CNS as its release was shown to be increased as a result of short-term habituation to an olfactory stimulus in *Drosophila* [9]. The mechanism of action of a ketogenic diet appears to include an anti-inflammatory and glycolytic metabolism pathway and with that appears to be an anticonvulsant. Similar to seizures, chronic pain is postulated to be related to increased excitability of neurons [62]; therefore, it is reasonable to study effects of this ketogenic diet on habituation to pain. An fMRI study in healthy participants reported evidence for a role of dopamine in habituation to pain, based on use of the dopamine D2 receptor antagonist haloperidol [63].

A recent review focusing on the genetic and molecular changes involved in habituation in general illustrated the complexity of the mechanism of action and molecules involved in habituation. In this review, various cellular pathways were highlighted, and the identification of 258 genes were reported as possible targets for drugs [64]. From this perspective, future research could investigate the effects of many more candidate drugs and their effect on habituation (and sensitization) to pain. 

In conclusion, the mechanisms underlying habituation to pain are poorly understood and likely to be related to a complex set of pathways including those related to inflammation, immune responses, neurotrophins, and/or neurotransmission. Research should focus on which pathways and molecules are most dominant in order to target them specifically, and this then may result in major impact on habituation to pain. In addition, further research may include other chronic pain indications and pharmacological options targeting habituation to pain. 

For now, no diagnostic markers are available for the prediction of habituation to pain. In addition, it is unclear when reduced habituation is of clinical relevance. For example, is a decrease of 0.5 point versus a 1.0 point decrease on the VAS after repeated stimulation an indication of reduced habituation to pain? Overall, it is too early to state that habituation trajectories (the response pattern resulting from repeated painful stimulation) are linked to clinical outcomes and could be used as a diagnostic marker for the prediction of chronic pain. Specifically for migraine patients, Brighina and colleagues stated that lack of habituation to pain probably represents a more general marker of neural dysfunction, with overlap of migraine with other pathologies such as chronic pain and Parkinson’s disease [65]. 

## 5. Challenges in the Field

There are several challenges in the field of habituation to pain in chronic pain conditions. Importantly, chronic pain indications are very heterogeneous. In addition, even within each individual chronic pain condition, there might be age and sex differences and differences in medication use (e.g., [11,38,43]). Furthermore, the experimental pain paradigms used are very diverse, including different modalities (e.g., heat and electric), stimulation sites, and the measure of habituation. Moreover, the link to clinical outcomes and experience of (chronic) pain in the studies is limited. The baseline pain levels of patients could potentially affect habituation, but this has not yet been investigated, with the exception of one study reporting reduced habituation in patients with greater widespread pain [43]. Furthermore, the experimental pain paradigms used in the studies included in this systematic review are not necessarily clinical pain-provoking. The latter would be of interest for the field. However, in addition to these differences, it is still of interest to investigate whether the antinociceptive system(s) differ in patients with chronic pain as compared to pain-free subjects. It is hypothesized that several neuroplasticity changes have already occurred (e.g., central sensitization) in chronic pain patients, and these changes may contribute to reduced habituation to pain [6,66,67]. In conclusion, although it is a challenge to standardize experimental pain paradigms in relation to specific pain indications it should be given much more attention in future studies. This is needed to better understand general effects on habituation to pain in chronic pain.

## 6. Future Directions for Research

Future research in chronic pain patients may inform us on the robustness of differences in habituation to pain in chronic pain patients as compared to (for instance) healthy controls and its underlying mechanisms. Neural measures such as EEG and fMRI could be analyzed in more detail using, for example, multilevel models for increased understanding of habituation to pain in chronic pain patients. Currently, evidence linking the self-report (behavioral) and EEG or fMRI (neural) measures is limited.

A second point which can be concluded from our review is that most studies were based on small sample sizes (median = 19.5) and did not always include a control group. Hence, there is a need for larger, blinded studies (i.e., the assessor is blinded for the group), including control groups and randomized controlled trials for potential treatment effects. In addition, direct group comparisons are necessary to obtain more conclusive results. Our review showed that one group sometimes showed significant habituation, while the other group did not; hence, it was concluded that there was no difference between patients and controls. However, without directly comparing groups, this conclusion cannot be made [68]. 

In general, chronic pain indications are very heterogeneous, and this makes generalization of conclusions often very difficult. An alternative might be to focus on individual differences in habituation to pain. Studying individual characteristics and differences may result into a better understanding of the heterogeneity in both patients and controls, and these effects may then be linked to clinical outcomes. Current studies in chronic pain patients did not focus on individual differences or age- and sex-related differences. This, however, would be an interesting topic for further research as studies in healthy individuals pointed out large individual differences (e.g., [69]), but conflicting evidence for age and sex (e.g., [38,70,71,72]. Ideally, individual differences in habituation to pain could also be used in prediction models for chronic pain or treatment effects. Longitudinal designs might then help to unravel the role of habituation in (the transition to) chronic pain. Investigation of a surgical population as they may develop postoperative (chronic) pain is recommended [73].

**Figure 5:**
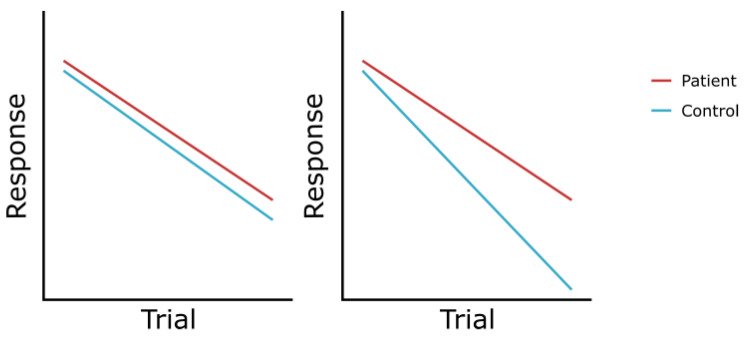
Example of patient groups that show similar habituation, whereas control groups differ in their response. With conventional analyses, panel one will result in no group differences whereas panel two will result in group differences, complicating the conclusion of patient vs. control effects.

An important issue in the correct analysis of studies on habituation to pain is the use and selection of statistical tests. In order to test and improve comparison of effects in studies on habituation to pain, we are in need of clear standardized measures to compare across studies and between patients and controls. Currently, there are several outcome measures for habituation to pain such as direct comparison of trials, linear effects (e.g., tested with a repeated-measures ANOVA), percentage change over averaged trials, habituation quotient (i.e., ratio between the average response in the first and last block), or fitting a (linear or quadratic) slope. With this variability in outcome measures, a standardized systematic comparison (such as in a meta-analysis) is not possible. Recently, recommendations for interpreting different habituation (to pain) patterns have been proposed [2]. With this, effects on habituation to pain might possibly be linked to phenotypes. On some occasions, it could be that patients show similar reduced habituation, but that the control group shows a different effect (see Figure 5), which is not captured in statistical tests. 

**Figure 6:**
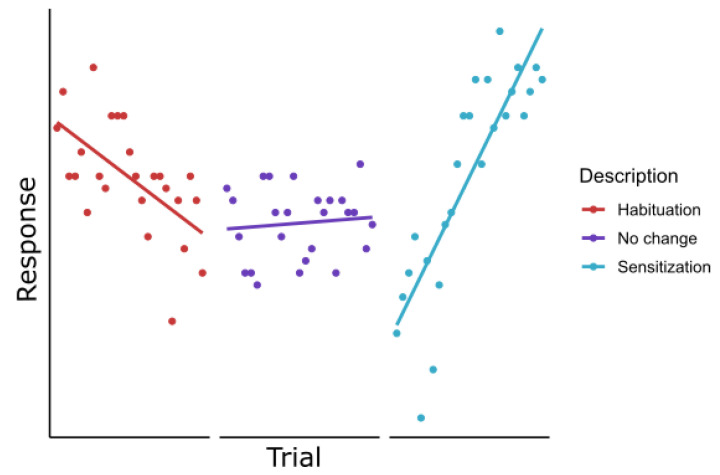
Example of data with a fitted (linear) slope that indicates the direction of the effect (after tested against zero), i.e., habituation, no change, or sensitization.

Therefore, we propose the fitting of a slope as indicator of the trajectory of the habituation (linear, quadratic, etc.). The use of these slopes and trajectory of habituation to pain has several advantages over current measures; it does not require any calculation of the dependent variable (such as averaging), it is easy interpretable and indicates the direction of effects, i.e., habituation or sensitization, and, when tested against zero, it can also indicate the significance of changes compared to zero (see Figure 6). However, it would require a fixed number of trials to be comparable across studies, and it can be influenced by other factors such as interstimulus interval and type of stimulation. This is in general of influence for habituation to pain, which is why the field will greatly benefit from standard setups and measures. If the field progresses to standard protocols and outcome measures as they are currently used in quantitative sensory testing (QST), it will be possible to increase the understanding of habituation to pain and its potential role and link to chronic pain [74,75]. In addition, recently developed statistical analysis methods, such as the high-temporal-resolution EEG analysis method and the event-related fixed-interval area method, are promising improvements in the detailed investigation of habituation of pain [38,76]. 

Furthermore, recommendations such as the use of standard terminology, comparison of similar outcome measures (i.e., not comparing EEG effects with rating effects), taking into account the use of different timescales when analyzing and interpreting the data, and taking into account individual differences will improve future study design and analyses. 

### Limitations

In this review, the effects of habituation to non-painful stimuli, the pain threshold, physiological measures such as skin conductance, and stimulation paradigms where the intensity was adjusted were not included (e.g., [77,78]). Thus, reduced habituation to pain in chronic pain patients may exist according to the use of different measures, and future research is needed to explore these measures. Furthermore, this review focused mainly on habituation to pain but not on sensitization to pain in chronic pain patients. These closely related processes should preferably be described and studied together, but most studies only deal with either habituation or sensitization to pain.

## 7. Conclusions

This review systematically summarized the available evidence on habituation to pain in different chronic pain indications. Although several studies reported reduced habituation to pain in migraine for self-report and the EEG-based N1 and N2–P2 amplitude, further evidence and confirmation based on larger, well-designed studies is needed. In CLBP patients, the evidence argues against any general differences, except for EEG measures. In fibromyalgia, there is evidence for reduced habituation to pain of the N2–P2 amplitude. Currently, the evidence of a diagnostic marker or linking habituation to pain to clinical outcomes is limited. Future studies should include standard outcome measures to improve the comparison of habituation to pain in chronic pain patients and controls. The mechanisms underlying habituation to pain are poorly understood and likely to be related to a complex set of pathways. Recent use of genetic and molecular analysis techniques allows for better understanding and selection of new pharmacological treatment options which then may help to reduce pain in chronic pain patients.

## Figures and Tables

**Table 2:** Overview of habituation to pain in CLBP. ↘: decrease over time (habituation). →: no significant change. ↗: increase over time (sensitization). <: reduced habituation in the chronic pain patient compared to the control group. =: no significant difference in habituation between the groups. BOLD, blood-oxygen-level-dependent; C, control; CLBP; chronic low back pain; EEG, electroencephalography; fMRI, functional magnetic resonance imaging; ISI, inter-stimulus interval, NRS, numeric rating scale; P, painful radiculopathy; VAS, visual analogue scale.

	Sample Size	Timescale	Type of Stimuli	Site	Nr of Stimuli for Habituation Analysis	Duration	ISI	Habituation Measurement	HabituationAnalysis	Main Habituation Result
**Arntz et al., 1991** [34]	CLBP = 22 C = 21	Short-term	Electrical	Thumb of left hand	20	1 s	15–45 s	VAS: pretest, last trial of blocks, post-test	Trend over time	CLBP ↘ = C ↘
**Hüllemann et al., 2017** [36]	Painful radiculopathy = 27 C = 20	Short-term	Heat (YAP laser)	Middle ventral thigh	100	5 ms	8–12 s	NRS: single trail EEG: N2–P2 amplitude	Trend over blocks	P ↘ = C ↘ P ↘ < C ↘
**Kersebaum et al., 2021** [38]	Painful radiculopathy *n* = 14 for twelve blocks and *n* = 18 for six blocks, controls *n* = 10 for twelve blocks and *n* = 14 for six blocks	Short-term	Heat (YAP laser)	Middle ventral thigh	100	5 ms	8–12 s	NRS: single trial EEG: N2–P2 amplitude	High-temporal-resolution analysis	NRS: over 12 blocks P ↘ C NA, over six blocks P → C → N2–P2: over 12 blocks P ↘ C NA, over six blocks P ↘ C →
**May et al., 2012** [10]	CLBP = 21 C = 66	Long-term (8 days)	Heat (thermode)	Left volar forearm	480	6 s	–	VAS: average rating of last 6 stimuli	Trend over time	Within-session: CLBP ↗ = C ↗ Between-session: CLBP ↘ = C ↘
**Peters et al., 1989** [35]	CLBP = 20 C = 20	Short-term	Pressure	Index finger of non-dominant hand	6	70% of pain tolerance time	4 min	VAS: single trials	Trend over time	CLBP → = C →
**Rodriguez-Raecke et al., 2014** [11]	CLBP = 19 C = 21	Long-term (8 days)	Heat (thermode)	Left volar forearm	480	6 s	–	VAS: average rating of last 6 stimulifMRI: BOLD	Trend over time Whole-brain contrast	Within-session: CLBP ↗ = C ↗ Between-session: CLBP ↘ = C ↘ CLBP = C
**Vossen et al., 2015** [37]	CLBP = 65 C = 76	Short-term	Electrical	Left middle finger	150	10 ms	9–11 s	EEG: amplitude	Multilevel model with event-related fixed-interval areas	CLBP ↘ < C ↘

**Table 3:** Overview of habituation to pain in fibromyalgia patients. <: reduced habituation in the chronic pain patient compared to the control group. =: no significant difference in habituation between the groups. C, control; EEG, electroencephalography; F = fibromyalgia; FMD; fibromyalgia with proximal and distal denervation; FMN, fibromyalgia with normal skin biopsy; FMP, fibromyalgia with proximal denervation; ISI, inter-stimulus interval; M = migraine; TMD, temporomandibular disorder; VAS, visual analogue scale.

	Sample Size	Timescale	Type of Stimuli	Site	Nr of Stimuli for Habituation Analysis	Duration	ISI	Habituation Measurement	HabituationAnalysis	Main Habituation Result
**de Tommaso et al., 2011** [41]	F = 14 C = 13	Short-term	Heat (CO_2_ laser)	Dorsum of the right hand, right supraorbital zone and knee	20	25 ms	10 s	VAS: average per block EEG: N1, N2, P2, and N2–P2 amplitude	Quotient between third and first block	VAS: F < C N1: F = C N2, P2, and N2–P2: F < C No differences between sites Self-reported depressive symptoms correlate with N2 habituation
**de Tommaso et al., 2014** [42]	F combined = 199 F = 94 F with M = 70F with sensory deficits = 35 C = 109	Short-term	Heat (CO_2_ laser)	Dorsum of the right hand, chest and knee	10	30 ms	10 s	EEG: N2–P2 amplitude	Quotient between third and first block	All sites: F combined < C F < C F with M < C F with sensory deficits < C F with M < F F with M < F with sensory deficits No correlation between habituation and self-reported depressive symptoms
**de Tommaso et al., 2017** [43]	F = 50 C = 30	Short-term	Heat (CO_2_ laser)	Dorsum of the right hand and foot	30	30 ms	10 s	EEG: N2–P2 amplitude	Percent amplitude change between third and first group of responses	Hand and foot: F < C
**Hollins et al., 2011** [40]	F = 17 TMD = 29 C = 29	Short-term	Heat (thermode)	Base of the thumb	33	3 s	12 s	VAS: single trial	Decrease over blocks	F = TMD = C
**McLoughlin et al., 2011** [45]	F = 16 C = 18	Short-term	Heat (thermode)	Left hand palm	5	20 s	20 s	VAS: single trial	Difference score	Self-reported activity correlated negatively with pain and unpleasantness difference scores in patients
**Vecchio et al., 2020** [46]	F = 81	Short-term	Heat (CO_2_ laser)	Dorsum of the right hand, in subgroups also thorax and dorsum of the foot	30	30 ms	10 s	EEG: N2–P2 amplitude	Ratio between third and first block	Thigh: habituation index of N2–P2 predicted intra-epidermal nerve fiber density
**Vecchio et al., 2022** [44]	F = 41 (F with normal skin biopsy FMN = 18, F with proximal denervation FMP = 22, F with proximal and distal denervation FMD = 7) C = 15	Short-term	Heat (CO_2_ laser)	Thigh and dorsum of the foot	30	30 ms	10 s	EEG: N2 and P2 amplitude	Change over time between third and first block	Thigh N2: F = C Thigh P2: F < C (all groups, FMN > FMP, FMD) Foot P2: F = C Patients with reduced intra-epidermal nerve fiber density showed less habituation of the P2 component

## Data Availability

No new data were created or analyzed in this study. Data sharing is not applicable to this article.

## Supplementary Materials

The following supporting information can be downloaded at https://www.mdpi.com/article/10.3390/jcm12134305/s1: Material and Methods; Figure S1. Flow diagram of selection and inclusion; Table S1: Inclusion and exclusion criteria.
