# Supplementary material for: Habituation to Pain in Patients with Chronic Pain: Clinical Implications and Future Directions
Source: J Clin Med. 2023 Jun 27;12(13):4305. doi: 10.3390/jcm12134305 (PMC10342770; doi:10.3390/jcm12134305)
Supplement: Supplementary file 1 [file jcm-12-04305-s001.zip › jcm-2430390-supplementary.pdf]

Material and Methods

Search strategy

PubMed, PsycINFO and Web of Science databases were searched for eligible studies from database initiation until July 2020. Search terms consisted of elements related to pain AND habituation (see below for the full strategy of PubMed). Terms related to “repeated painful stimulation” were also added, to ensure a full inclusion of studies where habituation might be investigated but not explicitly termed or comparable terms were used. No filters were applied. Reference lists of articles fulfilling the inclusion criteria were manually screened for additional references. Prior to finalizing the manuscript, a final search was performed including all articles published until January 2023.

Study criteria

Exclusion criteria were preregistered at the Open Science Framework (osf.io/nypbw) and evaluated in order (see Table 1; inclusion criteria are added for clarity). This review focuses on self-report ratings and brain measures (EEG and fMRI) of habituation to pain. Consequently, articles should include one of these three measures and describe a habituation process involving repeated painful stimulation at the same intensity. Studies that included paradigms where the stimulation intensity was adapted as a measure of habituation were not included. Studies on habituation of pain thresholds were also excluded because they do not require repeated stimulation above the pain threshold, which is our primary interest and involves a different evaluation process (e.g., decision-making whether something is painful and how much pain can be tolerated).

Selection procedure

References from PubMed, PsycINFO and Web of Science databases were combined in EndNote and de-duplicated (see Figure 1). Titles and abstracts were screened for eligibility by two independent reviewers (MvdM and CV). Full-text screening was performed as a final selection round for all articles that were likely relevant or unclear based on the title and abstract only. This was performed by the same reviewers (MvdM and CV). This led to the inclusion of N=40 articles (see Figure 1). This review was originally preregistered to include both healthy participants and chronic pain patients. However, due to abundant available literature, the review was eventually split in one part about healthy participants and the current part about habituation to pain in chronic pain patients.

Criterion	Included	Excluded
**1. Article type**	Research manuscripts	Review, commentary, editorial, thesis, book section, meta-analyses
**2. Population**	Adults	Animals, children
**3. Paradigm**	Painful repeated stimulation related to habituation	No painful stimuli, visual stimuli, auditory stimuli, unrelated paradigm (e.g., survey, sensitization)
**4. Response measurement**	Multiple pain ratings or neural activity	Measures of behavioral habituation, blood pressure, pain threshold, reflex, skin conductance
**5. Language**	English	Other
**6. Imaging**	EEG and fMRI	Studies without functional imaging during the habituation process and PET, sMRI, fNIRS, DTI

**7. Intervention**	Factors influencing habituation	Habituation effects of interventions, e.g., electrical nerve stimulation, spinal cord stimulation)
**8. Other**		Intracranial recordings, n = 1, full text not available

Table S1. In- and exclusion criteria.

Abbreviations: DTI, diffusion tensor imaging; EEG, electroencephalography; fMRI, functional magnetic resonance; fNIRS, functional near-infrared spectroscopy; PET, positron emission tomography; sMRI, structural magnetic resonance imaging.

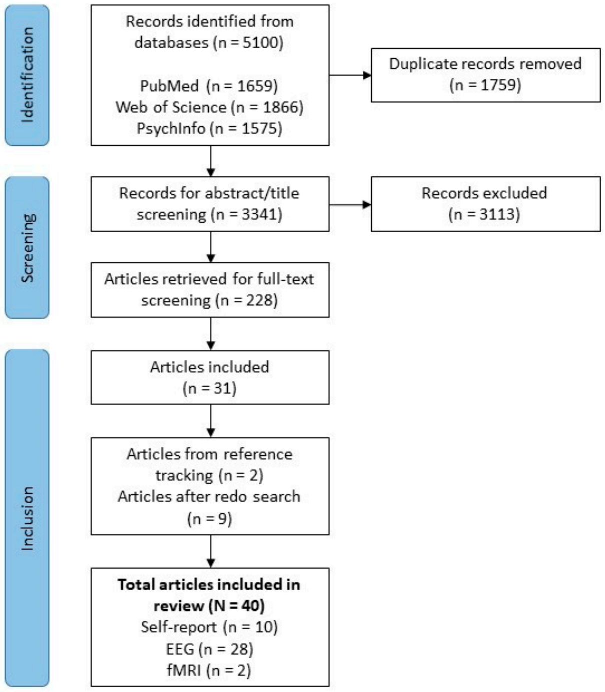

Figure S1. Flow diagram of selection and inclusion.

Full search strategy for PubMed database

((("Chronic Pain"[Mesh] OR chronic Pain*[tiab] OR "Pain"[Mesh] OR pain*[tiab] OR "Pain Threshold"[Mesh] OR pain threshold*[tiab] OR ache*[tiab] OR physical suffering*[tiab] OR

arthrosis[tiab] OR arthropathy*[tiab] OR arthritis[tiab] OR cephalic syndrome*[tiab] OR contracture*[tiab] OR chondropath*[tiab] OR cephalgia*[tiab] OR cephalea*[tiab] OR cephalalgia*[tiab] OR "Colonic Diseases, Functional"[Mesh] OR colon disease*[tiab] OR colonic disease*[tiab] OR Crohn*[tiab] OR colitis[tiab] OR colon diverticulosis[tiab] OR colon fistula*[tiab] OR colorectal disease*[tiab] OR "Endometriosis"[Mesh] OR endometriosis[tiab] OR endometrioses[tiab] OR endometrioma[tiab] OR enthesopath*[tiab] OR fibrositis[tiab] OR fibromyalgia[tiab] OR "Fibromyalgia"[Mesh] OR functional colonic disease*[tiab] OR “Headache Disorders"[Mesh] OR headache*[tiab] OR ischialgia[tiab] OR irritable bowel syndrome*[tiab] OR irritable colon*[tiab] OR irritable colon syndrome*[tiab] OR IBD[tiab] OR inflammatory bowel disease*[tiab] OR migraine*[tiab] OR mononeuropath*[tiab] OR musculoskeletal disease*[tiab] OR myalgia[tiab] OR nerve fiber inflammation*[tiab] OR nerve compression*[tiab] OR nerve entrapment*[tiab] OR nervous entrapment*[tiab] OR nerve constriction*[tiab] OR nerve injur*[tiab] OR neuralg*[tiab] OR neuritis[tiab] OR neuropath*[tiab] OR osteoarthrosis[tiab] OR osteoarthritis[tiab] OR phantom limb*[tiab] OR "Peripheral Nervous System Diseases"[Mesh] OR peripheral nervous system Disease*[tiab] OR PNS disease*[tiab] OR peripheral nerve disease*[tiab] OR peripheral nervous disease*[tiab] OR peripheral nervous system Disorder*[tiab] OR PNS disorder*[tiab] OR peripheral nerve disorder*[tiab] OR peripheral nervous disorder*[tiab] OR polyneuritis[tiab] OR polyradiculoneuritis[tiab] OR polyneuropath*[tiab] OR radiculoneuritis[tiab] OR radiculopath*[tiab] OR rheumatic[tiab] OR rheumatism[tiab] OR Spondyloarthritis[tiab] OR spondylarthritis[tiab] OR spondylitis[tiab] OR Sciatica[tiab])))

AND ("Habituation, Psychophysiologic"[MeSH] OR habituat*[tiab] OR repeated stim*[tiab] OR repeated pain*[tiab] OR repeated noxious[tiab] OR repetitive pain*[tiab] OR repetitive noxious[tiab] OR repetitive stim*[tiab])
